# Supplementary material for: Dynamical control of quantum heat engines using exceptional points
Source: Nat Commun. 2022 Oct 20;13:6225. doi: 10.1038/s41467-022-33667-1 (PMC9584956; doi:10.1038/s41467-022-33667-1)
Supplement: Supplementary file 1 — Supplementary Information [file 41467_2022_33667_MOESM1_ESM.pdf]

Supplementary materials for
"Dynamic Control of Quantum Heat Engines with Exceptional Points"

J.-W. Zhang, J.-Q. Zhang, G.-Y. Ding, J.-C. Li, J.-T. Bu, B. Wang, L.-L. Yan,
S.-L. Su, L. Chen, F. Nori, S. K. Özdemir, F. Zhou, H. Jing, M. Feng

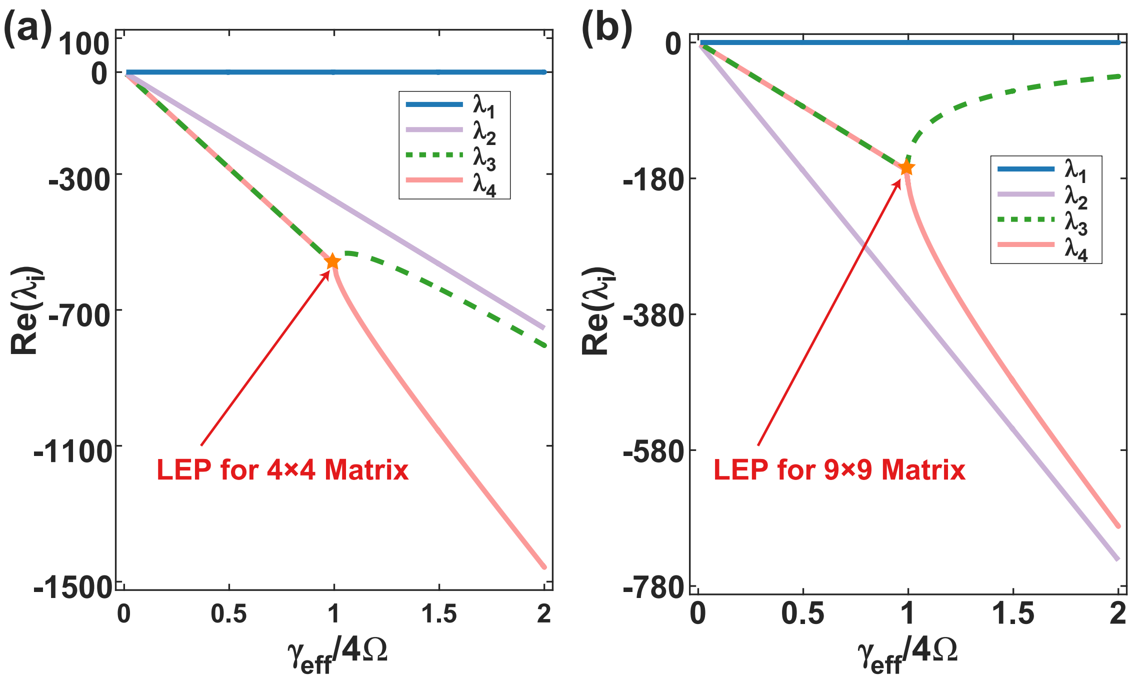

Supplementary Figure 1: Comparison of the real parts of the eigenvalues $\lambda_{3,4}$ between a 4×4 matrix (i.e., effective two-level case) and a 9×9 matrix (i.e., three-level case) with respect to $\gamma_{\rm eff}/4\Omega$. We see the bifurcations (denoted by orange stars) in both panels occur at $\gamma_{\rm eff}/4\Omega = 1$, indicating that the two cases share the same Liouvillian exceptional point (LEP). The horizontal blue line shows λ_1 and the pink straight line indicates λ_2, but both are irrelevant to our experimental observation. For the case of 9×9 matrix, we only plot four eigenvalues for comparison, and neglect the remaining unphysical eigenvalues.

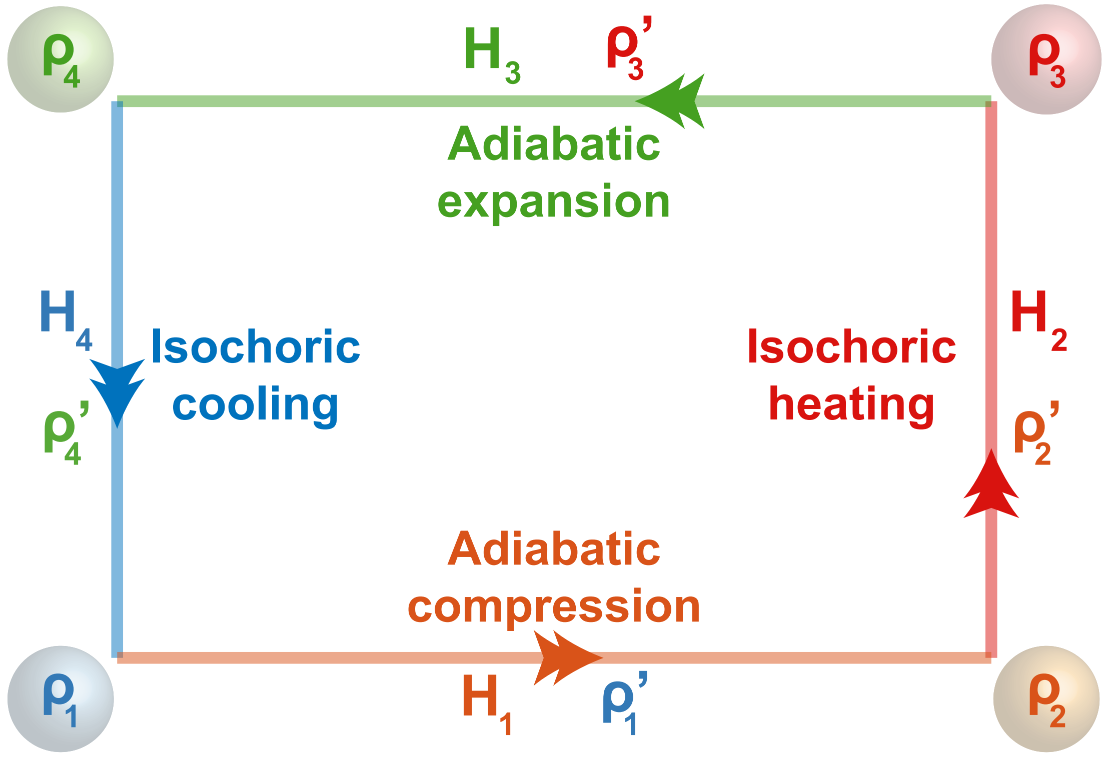

Supplementary Figure 2: A quantum Otto cycle is composed of four strokes: two adiabatic strokes referred to as adiabatic compression and expansion and two isochoric strokes describing the energy exchange with a hot and cold bath. The density operators remain constant during adiabatic strokes, that is $\rho_2 = \rho_1$, and $\rho_3 = \rho_4$. The density operators change during the isochoric strokes, implying energy exchange with either the hot or cold bath. H_i denotes the Hamiltonian describing the ith stroke, and ρ_i' represent the time-dependent matrix in the ith Stroke.

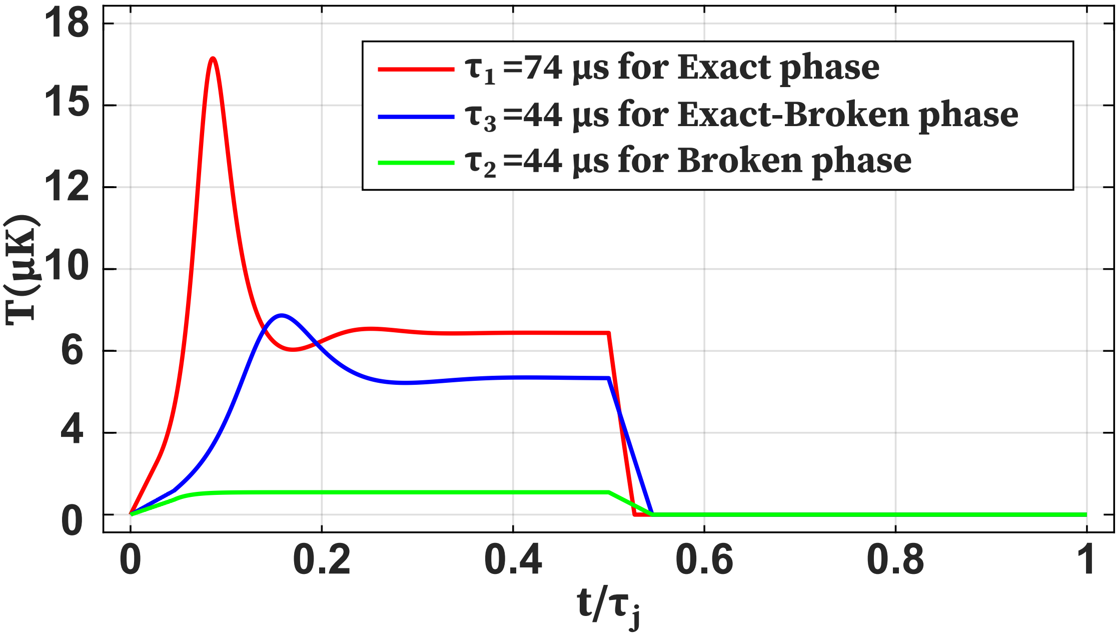

Supplementary Figure 3: Time evolution of the effective temperature T_{eff} in a quantum Otto cycle in the single ion heat engine. The red and green curves represent, respectively, the Otto cycle with both isochoric processes in the exact phase and both in the broken phase. The blue curve represents the isochoric heating process in the exact phase but the isochoric cooling process in the broken phase. τ_i is the total execution time of the Otto cycle.

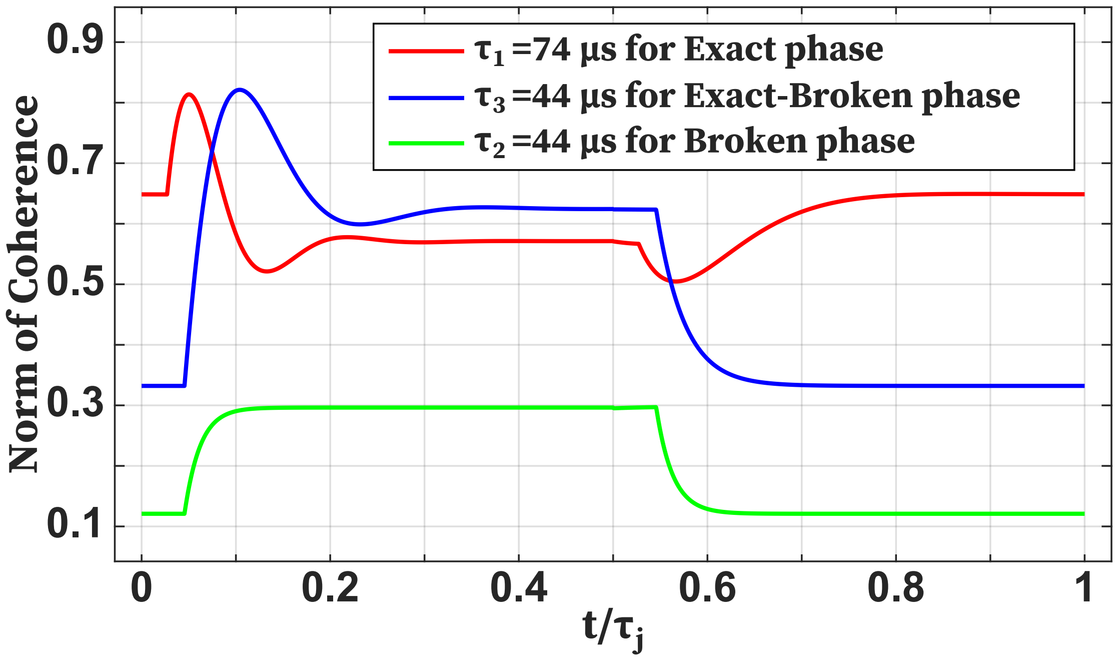

Supplementary Figure 4: The evolution of coherence in a quantum Otto cycle in the trapped single ion heat engine. The red and green curves represent, respectively, the Otto cycle with both isochoric processes are implemented in the exact phase and in the broken phase. Blue curve represents the case where the isochoric heating process is implemented in the exact phase and the isochoric cooling process implemented in the broken phase. τ_i is the total execution time of the Otto cycle.

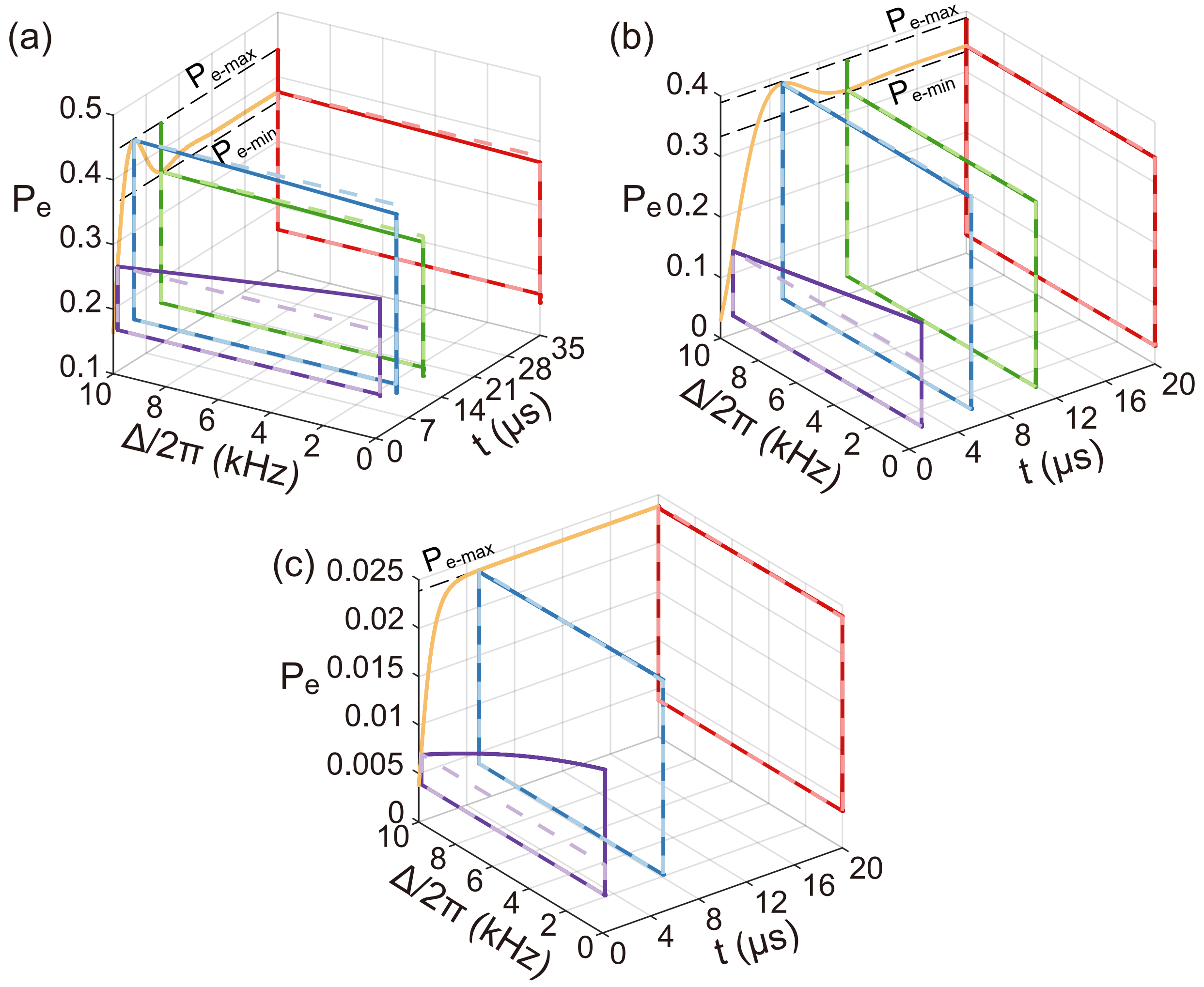

Supplementary Figure 5: Dynamical evolution of the population versus the duration of the isochoric heating stroke. (a), (b) and (c) represent, respectively, the Otto cycle with isochoric processes in exact phase, with the isochoric heating process in exact phase but the isochoric cooling process in broken phase, and with isochoric processes in broken phase. The dashed lines correspond to the ideal evolution of the population and the solid lines represent numerical simulation using experimentally available parameter values.

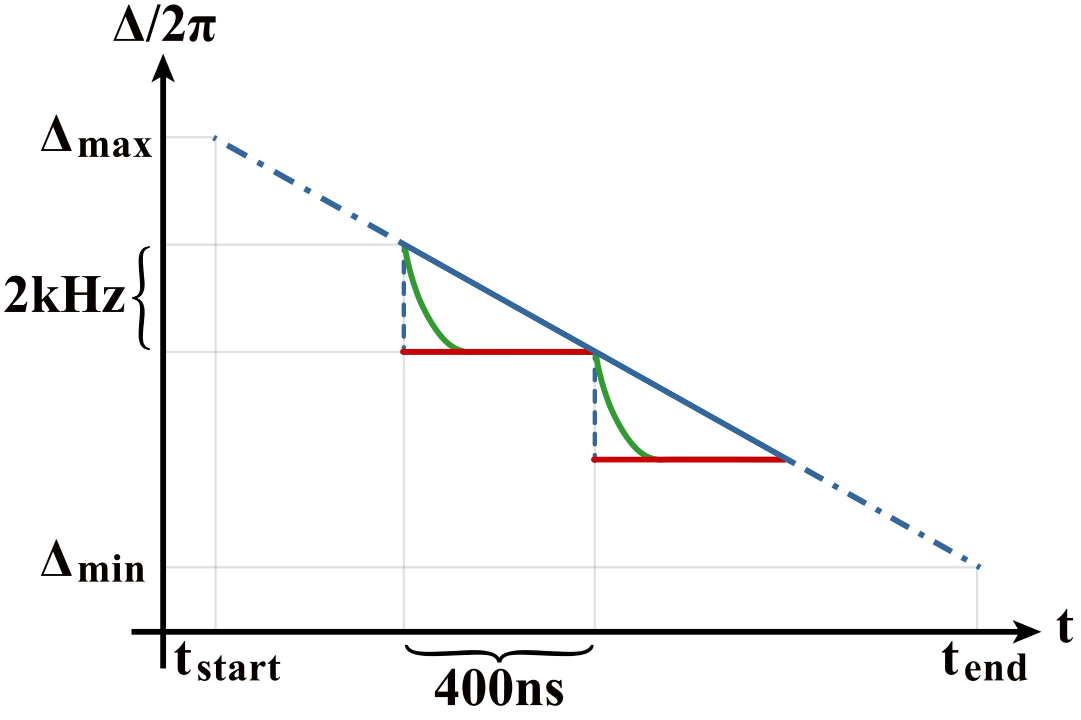

Supplementary Figure 6: Schematic for the sequential change of Δ in the adiabatic expansion stroke. Ideally, the detuning Δ should be tuned smoothly in a continuous fashion (blue line). In the experiments; however, this is approximated by a sequence of discrete steps where each step decreases the detuning by 2 kHz within 400 ns (red line) using an acousto-optic modulator (AOM). This results in a tuning curve (green) which is slightly different than the ideal tuning curve (blue), leading to unexpected phases in the operation of our system.

Supplementary Note 1: Hamiltonian and Effective decay rate

The Hamiltonian describing the three-level system which corresponds to the single trapped ion in our experiments is given as:

$$H_s = \omega_e \left|e\right\rangle \left\langle e\right| + \omega_p \left|p\right\rangle \left\langle p\right| + \tfrac{\Omega}{2}(\left|e\right\rangle \left\langle g\right| e^{-i\omega_l t} + \left|g\right\rangle \left\langle e\right| e^{i\omega_l t}) + \tfrac{\Omega_p}{2}(\left|e\right\rangle \left\langle p\right| e^{i\omega_d t} + \left|p\right\rangle \left\langle e\right| e^{-i\omega_d t}), \tag{1}$$

where ω_p and ω_e are, respectively, the energies of the levels $|p\rangle$ and $|e\rangle$ with respect to the level $|g\rangle$, and ω_l and ω_d are, respectively, the frequencies of the lasers coupling $|g\rangle$ to $|e\rangle$ and $|e\rangle$ to $|p\rangle$. This Hamiltonian H_s satisfies the Schrödinger equation (in units of $\hbar = 1$)

$$\frac{d}{dt}\left|\psi\right\rangle_s = -iH_s\left|\psi\right\rangle_s, \tag{2}$$

where $H_s = H_0 + H_1(t)$, with H_0 corresponding to the time-independent part of the Hamiltonian. Then defining $|\psi\rangle_s = U_I |\psi\rangle_I$ with $U_I = e^{-iH_0 t}$, we rewrite the Schrödinger equation and obtain

$$U_I^\dagger U_I \frac{d}{dt}\left|\psi\right\rangle_I = -i\Big(U_I^\dagger H_s U_I - iU_I^\dagger \frac{dU_I}{dt}\Big)\left|\psi\right\rangle_I = -iH_I\left|\psi\right\rangle_I, \tag{3}$$

which implies that the Hamiltonian H_I in the interaction picture satisfies

$$H_I = U_I^\dagger H_s U_I - iU_I^\dagger \tfrac{dU_I}{dt} = U_I^\dagger H_s U_I - H_0. \tag{4}$$

Defining $H_0 = \omega_l \left|e\right\rangle \left\langle e\right| + \omega_p \left|p\right\rangle \left\langle p\right|$ and using the Baker-Hausdorff formula

$$e^{iH_0 t} A e^{-iH_0 t} = A + it[H_0, A] + \frac{(it)^2}{2!}[H_0, [H_0, A]] + \cdots \tag{5}$$

we find

$$H_I \;=\; \Delta \left|e\right\rangle \left\langle e\right| + \tfrac{\Omega}{2}(\left|e\right\rangle \left\langle g\right| + \left|g\right\rangle \left\langle e\right|) + \tfrac{\Omega_p}{2}(\left|e\right\rangle \left\langle p\right| + \left|p\right\rangle \left\langle e\right|), \tag{6}$$

where we have $\Delta = \omega_e - \omega_l$ and $\omega_p = \omega_d + \omega_l$, $\omega_d = \omega_p - \omega_l = \omega_p - (\omega_e - \Delta) \simeq \omega_p - \omega_e$. The effective Hamiltonian H_{eff} is then given by [1]

$$H_{\text{eff}} = -\tfrac{1}{2}V_-[H_{\text{NH}}^{-1} + (H_{\text{NH}}^{-1})^\dagger]V_+ = \Delta \left|e\right\rangle \left\langle e\right| + \tfrac{\Omega}{2}(\left|e\right\rangle \left\langle g\right| + \left|g\right\rangle \left\langle e\right|), \tag{7}$$

where the non-Hermitian Hamiltonian H_{NH} is given by $H_{\text{NH}} = -\frac{i}{2}(\gamma_e + \gamma_g)\left|p\right\rangle \left\langle p\right| = -\frac{i}{2}\gamma \left|p\right\rangle \left\langle p\right|$, with γ_i corresponding to the decay rate from the level $|p\rangle$ to the level $|i\rangle$, while the perturbative excitations V_+ and de-excitations V_- are defined as $V_+ = \frac{\Omega_p}{2}\left|p\right\rangle \left\langle e\right| + \frac{\Omega}{2}\left|e\right\rangle \left\langle g\right|$ and $V_- = \frac{\Omega_p}{2}\left|e\right\rangle \left\langle p\right| + \frac{\Omega}{2}\left|g\right\rangle \left\langle e\right|$. We then write the effective Lindblad operator as [1]

$$L_{\text{eff}}^{e\to g} = i\sqrt{\gamma_g}\frac{\Omega_p}{\gamma}\left|g\right\rangle \left\langle e\right|, \tag{8}$$

from which the effective decay rate γ_{eff} from the excited state $|e\rangle$ to the ground state $|g\rangle$ is found as

$$\gamma_{\text{eff}} = \frac{\gamma_g \Omega_p^2}{\gamma^2} = \frac{(\gamma - \gamma_e)\Omega_p^2}{\gamma^2} = \Big(1 - \frac{\gamma_e}{\gamma}\Big)\frac{\Omega_p^2}{\gamma} \simeq \frac{\Omega_p^2}{\gamma}. \tag{9}$$

The same result by an alternative method can be found in [2]. We note that the expression for H_{eff} in Eq. (7) is the same as Eq. (2) in the main text and γ_{eff} appears in Eq. (1) of the main text and in the expression of the LEP of the system.

Supplementary Note 2: Liouvillian Exceptional Points

Our system obeys the Lindblad master equation $\dot{\rho}(t) = \mathcal{L}\rho(t)$ as defined in Eq. (1) of the main text. Here

$\mathcal{L}$ is the Liouvillian superoperator and ρ is the density operator of the system. From the Hamiltonian, as written in Eq. (7) and Eq. (2) of the main text, we can write $\mathcal{L}$ as

$$\mathcal{L} = \begin{pmatrix} -\gamma_{\text{eff}} & i\Omega/2 & -i\Omega/2 & 0 \\ i\Omega/2 & -(\gamma_{\text{eff}}/2 + i\Delta) & 0 & -i\Omega/2 \\ -i\Omega/2 & 0 & -(\gamma_{\text{eff}}/2 - i\Delta) & i\Omega/2 \\ \gamma_{\text{eff}} & -i\Omega/2 & i\Omega/2 & 0 \end{pmatrix}$$

By setting $\Delta = 0$, we find the eigenvalues of $\mathcal{L}$ as $\lambda_1 = 0$, $\lambda_2 = -\gamma_{\text{eff}}$, $\lambda_3 = \frac{1}{4}(-3\gamma_{\text{eff}} - \sqrt{\gamma_{\text{eff}}^2 - 16\Omega^2})$, and $\lambda_4 = \frac{1}{4}(-3\gamma_{\text{eff}} + \sqrt{\gamma_{\text{eff}}^2 - 16\Omega^2})$.

Since these eigenvalues are related to dissipation terms, their real parts indicate decaying effects while their imaginary parts define the eigenenergies [3]. It is easily seen that the eigenvalues λ_3 and λ_4 become degenerate and an LEP emerges at $\lambda_3 = \lambda_4 = -3\gamma_{\text{eff}}/4$, when $\gamma_{\text{eff}} = 4\Omega$. As explained in the main text, for $\gamma_{\text{eff}} > 4\Omega$, we have real λ_3 and λ_4 with a splitting amount given by $\xi = \sqrt{\gamma_{\text{eff}}^2 - 16\Omega^2})$. Thus, when $\gamma_{\text{eff}} > 4\Omega$, the system is in the broken phase. For $\gamma_{\text{eff}} < 4\Omega$, on the other hand, λ_3 and λ_4 become complex conjugate pairs with a splitting of ξ in their imaginary parts, corresponding to the exact phase.

Moreover, to justify the validity of our reduced matrix, i.e., Eq. (12), which comes from the effective Hamiltonian Eq. (8), we have calculated the LEPs for both Hamiltonians (6) and (7), and found that the LEPs share the same decay rate as written in (9) in the case of $\Delta = 0$. This is further confirmed in Supplementary Figure 1. The eigenvectors corresponding to the above eigenvalues are,

$$\rho_1 = \begin{pmatrix} \frac{\Omega^2}{\gamma_{\text{eff}}^2+\Omega^2} & -i\frac{\gamma_{\text{eff}}\Omega}{\gamma_{\text{eff}}^2+\Omega^2} \\ i\frac{\gamma_{\text{eff}}\Omega}{\gamma_{\text{eff}}^2+\Omega^2} & 0 \end{pmatrix}, \quad \rho_2 = \begin{pmatrix} 0 & 1 \\ 1 & 0 \end{pmatrix}, \tag{10}$$

and

$$\rho_{3,4} = \begin{pmatrix} -1 & -\frac{-i\gamma_{\text{eff}} \pm \sqrt{16\Omega^2 - \gamma_{\text{eff}}^2}}{4\Omega} \\ \frac{-i\gamma_{\text{eff}} \pm \sqrt{16\Omega^2 - \gamma_{\text{eff}}^2}}{4\Omega} & 1 \end{pmatrix}, \tag{11}$$

Clearly, at $\Delta = 0$ and $\gamma_{\text{eff}} = 4\Omega$, the eigenstates ρ_3 and ρ_4 coalesce, confirming that a second-order LEP emerges at $\gamma_{\text{eff}} = 4\Omega$, separating the exact- and broken-phases.

Supplementary Note 3: Thermodynamic quantities

In this section, we present the definitions and discussions on the five thermodynamic quantities [4]: the net work W, the output power P, and the efficiencies η_o, η_c, η_q. We note that in our system of a trapped ion, the internal energy of the spin heat engine is defined as $U = \text{tr}(\rho H)$, where ρ and H are the density matrix and Hamiltonian of the spin system, respectively. Moreover, the Hamiltonian $H = \Delta |e\rangle\langle e|$, the coupling strength Ω, and the effective dissipation rate γ_{eff} are used to tune the system such that an Otto engine cycle is performed, as explained in the main text. Quantum coherence affecting the performance of the quantum Otto cycles in our system is investigated by tuning the execution time t_2.

The net work and output power

In classical thermodynamics, the first law of thermodynamics is expressed as $dU = dW + dQ$. In contrast, in quantum thermodynamics [4, 5], it is expressed as $dU = d(\text{tr}(\rho H)) = \text{tr}(\rho dH) + \text{tr}(H d\rho)$ where the work and heat in differential form are defined as $dW = \rho dH$ and $dQ = H d\rho$, respectively. Then the energy absorption Q_{in} from the hot bath and the energy dissipation Q_{out} to the cold bath can be written as $Q_{\text{in}} = \sum_i H_i d\rho'_i$ (for $d\rho'_i > 0$) and $Q_{out} = \sum_i H_i d\rho'_i$ (for $d\rho'_i < 0$), respectively (see Supplementary Figure 2). As we discussed in the main text, when Q_{in} and Q_{out} are calculated for our quantum Otto cycle, we see that during both of the isochoric processes the system absorbs and releases energy due to the quantum coherence involved. This is reflected in the oscillation observed in the populations of the excited and ground states. Thus, the net acquired work and the corresponding output power can be written, respectively, as

$$W = Q_{\text{in}} + Q_{\text{out}} = \sum_i H_i d\rho'_i, \tag{12}$$

and

$$P = W/(t_1 + t_2 + t_3 + t_4), \tag{13}$$

where t_i is the execution time of the i-th Stroke in the cycle.

Heat engine efficiencies

Ideal Otto engine efficiency η_o: In an ideal adiabatic process, the density matrix remains constant ($\rho_2 = \rho_1$, and $\rho_3 = \rho_4$), where ρ_2 and ρ_3 are the density matrices of the initial and final states of the isochoric heating process, respectively, and ρ_4 and ρ_1 are the density operators in the isochoric cooling process (See main text Fig. 1F).

In classical thermodynamics, the energy can be transferred only from a hot system to a cold one, and the energy flow is unidirectional. We can write the energy absorption from the hot bath during the isochoric heating stroke as

$$Q^o_{\text{in}} = \text{tr}[(\rho_3 - \rho_2)H_2], \tag{14}$$

and the energy release to the cold bath during the isochoric cooling stroke as

$$Q^o_{\text{out}} = \text{tr}[(\rho_4 - \rho_1)H_4], \tag{15}$$

where $H_2 = \Delta_{\max} |e\rangle \langle e|$ and $H_4 = \Delta_{\min} |e\rangle \langle e|$ are the Hamiltonians in the isochoric heating and cooling strokes, respectively. Here, $\Delta_{\min}$ and $\Delta_{\max}$ are the minimum and maximum detunings, respectively. So, the corresponding ideal Otto engine efficiency can be written as

$$\eta_o = 1 - \frac{Q^o_{\text{out}}}{Q^o_{\text{in}}} = 1 - \frac{\Delta_{\min}}{\Delta_{\max}}, \tag{16}$$

which is in the ideal limit ($\eta_o = 1$) when $\Delta_{\min} = 0$.

Conventional Otto engine efficiency η_c: The efficiency of the conventional Otto cycle is defined by

$$\eta_c = \frac{W}{Q_{\text{in}}} = 1 - \frac{Q_{\text{out}}}{Q_{\text{in}}}. \tag{17}$$

Quantum Otto engine efficiency η_q: In contrast to the classical counterpart, the heat absorption takes place during both the isochoric heating and cooling strokes of a quantum Otto cycle. To focus on the coherent effects in the isochoric heating process, we define η_q to be only regarding the heat absorption in the isochoric heating process:

$$\eta_q = \frac{W}{Q^q_{in}}, \tag{18}$$

where $W = H_2 d\rho'_2 + H_4 d\rho'_4 = \text{tr}[H_2(\rho_3 - \rho_2)] + \text{tr}[(H_4(\rho_1 - \rho_4)]$ is the work done and Q^q_{in} is the heat absorption

$$Q^q_{\text{in}} = (P^L_h - P^S_h)\Delta_{\max}, \tag{19}$$

with P^L_h and P^S_h denoting, respectively, the final steady state and initial populations in the isochoric heating process. Since there is no change in the state of the system during the adiabatic strokes, we have $\rho_1 = \rho_2 = \rho^S_h$, $\rho_3 = \rho_4 = \rho_h(t)$. Using this in the above expressions, we find η_q as

$$\eta_q = \frac{W(t)}{Q^q_{in}} = \frac{(\Delta P_e + P^L_h - P^S_h)(\Delta_{\max} - \Delta_{\min})}{(P^L_h - P^S_h)\Delta_{\max}} = \frac{(\Delta P_e + P^L_h - P^S_h)}{(P^L_h - P^S_h)}\left(1 - \frac{\Delta_{\min}}{\Delta_{\max}}\right) = \left(1 + \frac{\Delta P_e}{(P^L_h - P^S_h)}\right)\eta_o, \tag{20}$$

where we have defined the time-dependent population difference caused by the quantum coherence as $\Delta P_e = P_h(t) - P^L_h$. It is clear in Eq. (20) that η_q equals to the classical ideal Otto engine efficiency η_o, that is $\eta_q = \eta_o$ when $\Delta P_e = 0$. Similarly, we have $\eta_q > \eta_o$ for $\Delta P_e > 0$, and $\eta_q < \eta_o$ for $\Delta P_e < 0$. Since ΔP_e is a signature of the coherence in the system, we conclude that coherence may increase or decrease the efficiency of a quantum Otto cycle.

Supplementary Note 4: Effective temperature and Coherence

In the interaction picture, we consider the two-level system consisting of the ground state $|g\rangle$ and the excited state $|e\rangle$, with energy gap Δ. The occupation probabilities P_e and P_g obey the Boltzman distribution $P_e/P_g = \exp[-\beta\Delta(t)]$, and $P_e + P_g = 1$, giving the effective temperature [4, 5]

$$T_{\text{eff}} = \frac{1}{k_B\beta} = \frac{\Delta}{k_B}\left(\ln\frac{P_g}{P_e}\right)^{-1}. \tag{21}$$

Supplementary Figure 3 depicts the evolution of the effective temperature during a quantum Otto cycle. Here the changes in the temperature are mainly due to the detuning variation in the adiabatic compression and expansion strokes and the population oscillation in the isochoric strokes. The effective temperature can be made zero if the detuning is set to zero.

Using the expression $C_{l_1}(\rho) = \sum_{i\neq j}|\rho_{i,j}|$ for the coherence [6], we have calculated the coherence involved in an Otto cycle in our system and presented the results in Supplementary Figure 4. We see that the coherence remaining at the end of an Otto cycle is the lowest when the system is operated such that both of the isochoric strokes are in the broken phase and the highest when both of the isochoric strokes are implemented in the exact phase.

Supplementary Note 5: Dynamical evolution of the population

We represent the dynamic evolution of the population in Supplementary Figure 5, in which more cycles are plotted for the three cases considered in our experiment, i.e., both the isochoric processes in the exact phase, the isochoric heating process in the exact phase but the isochoric cooling process in the broken phase, and both the isochoric processes in the broken phase. This figure helps to understand the variation of some key quantities in the heat engine performance.

Supplementary Note 6: Experimental details

During the implementation of the Otto cycles, we drive the qubit by an ultra-stable narrow linewidth laser with wavelength 729-nm as expressed in Eq. (2) of the main text. This driving laser is controlled by a double-pass acousto-optic modulator which helps control the phase and the frequency of the 729-nm laser. We use a field programable gate array to control a direct digital synthesizer as the frequency source of the acousto-optic modulator. We repeat each single-qubit measurement 10,000 times to minimise the quantum projection noise. Due to the noises caused by the fluctuations in the applied magnetic and electric fields, the qubit in our system suffers from dephasing of 0.81(11) kHz. Other sources of error in our experiments are the laser instability and imperfections in the single-qubit pulses, whose effects are assessed from the Rabi oscillations. After calibration, we estimate the total error in the initial-state preparation and the final-state detection to be 0.7(2)% and 0.22(8)%, respectively. The influence of these noises and imperfections are reflected in the error bars shown in the figures of the main text.

In Figs $2A_1$, $2B_1$ and 3A, we note a slight reduction ($\approx$0.1) in the population of the excited state $|e\rangle$ during the first and third strokes. This deviation from the ideal theoretical expectation (i.e., no population change during the adiabatic compression and expansion strokes) can be attributed to the fact that in the experiments the frequency of the 729 nm laser was not tuned smoothly in a continuous fashion but instead we used a sequence of discrete steps using an AOM. As shown in Supplementary Figure 6, although the required continuous change of the detuning Δ (blue line) can be equivalently accomplished by discrete steps of operation, as plotted by the red line. In our experiments, the variation of detuning is accomplished by a sequence of AOM which leads to a slightly different tuning curve (green) due to the switching time of the AOM. This leads to imperfect variation of the detuning, bringing in unexpected phases in the operation. Nevertheless, considering these unexpected phases in numerical simulation, we have fitted the experimental observations involving such cases by theory very well, see Figs. $2A_1$, $2B_1$ and 3A in the main text.

[1] Reiter, F., & Sϕrensen, A. S. Effective operator formalism for open quantum systems, *Phys. Rev. A.* **85**, 032111 (2019).
[2] Zhang, J. W., Rehan, K., Li, M., Li, J. C., Chen, L., Su, S. L., Yan, L. L., Zhou, F., & Feng, M. Single-atom verification of the information-theoretical bound of irreversibility at the quantum level, *Phys. Rev. Research* **2**, 033082 (2020).

[3] Minganti, F., Miranowicz, A., Chhajlany, R. W., & Nori, F. Quantum exceptional points of non-Hermitian Hamiltonians and Liouvillians: The effects of quantum jumps, *Phys. Rev. A* **100**, 062131 (2019).

[4] Quan, H. T., Liu, Y. X., Sun, C. P., & Nori, F. Quantum thermodynamic cycles and quantum heat engine, *Phys. Rev. E* **76** 031105 (2007).

[5] Benenti, G., Casati, G., Saito, K., & Whitney, R. S. Fundamental aspects of steady-state conversion of heat to work at the nanoscale, *Phys. Rep.* **694**, 1 (2017).

[6] Baumgratz, T., Cramer, M., & Plenio, M. B. Quantifying Coherence, *Phys. Rev. Lett.* **113**, 140401 (2014).
